# Atopic dermatitis and melanoma: A case-control study

**DOI:** 10.1186/2049-3258-73-S1-P1

**Published:** 2015-09-17

**Authors:** Vivien Marasigan, Marie-Anne Morren, Julien Lambert, Karen Medaer, Steffen Fieuws, Marjan Garmyn

**Affiliations:** 1 University Hospitals Leuven, Leuven, Belgium; 2 Antwerp University Hospital, Antwerp, Belgium; 3 AZ Sint Jozef, Malle, Belgium; 4 Catholic University Leuven, Leuven, Belgium

## Background

Atopic dermatitis (AD) has been linked to fewer nevi which implies a decreased risk for melanoma. Yet scarce studies have been conducted on the association between AD and melanoma.

## Aim

To analyze the association of AD and melanoma development in a case-control population.

## Methods

A cohort of cutaneous melanoma patients treated at the Department of Dermatology in University Hospitals Leuven and Antwerp University Hospital in Belgium were included. Cases and randomly selected controls were interviewed. The U.K. Working Party's diagnostic criteria for AD was utilized in the ascertainment of AD. Cases were matched with controls on important confounders and gender. In addition to AD, personal atopy, respiratory atopy, active atopy and familial atopy, and their relation to melanoma development, were also investigated. Statistical analyses were performed using Mc Nemar's test and conditional logistic regression.

## Results

188 cases and 596 controls were included. Analysis showed a general inverse association between atopy related outcome variables and melanoma development but only personal atopy was statistically significant (OR=0.53,CI:0.30-0.96,p-value=0.04). A separate analysis showed that nevi count was the most important confounder. Therefore, a sub-analysis was conducted in the group with no or few moles whereby personal atopy remained statistically significant (p-value=0.03).

## Conclusion

This is a case-control study on the association between AD and melanoma using a valid AD questionnaire, suggesting an inverse association between personal atopy and melanoma when matched for important confounders. However, no strong claim can be made when corrected for multiple testing.

